# Evolutionary Origins and Virulence Determinants of ST25 Hypervirulent *Klebsiella pneumoniae* in Swine: Genomic Insights and Functional Validation

**DOI:** 10.1155/tbed/4488875

**Published:** 2026-01-21

**Authors:** Zheng Chen, Yang Liu, Congyang Du, Shengguo Gao, Lufeng Zhai, Stefan Schwarz, Weicheng Liu, Qiong Li, Longyu Li, Runhao Yu, Yuzhe Zhao, Hong Yao, Lei Luo, Xue Liu, Chunyan Xu, Xiang-Dang Du

**Affiliations:** ^1^ International Joint Research Center of National Animal Immunology, Ministry of Education Key Laboratory for Animal Pathogens and Biosafety, College of Veterinary Medicine, Henan Agricultural University, Zhengzhou, 450046, China, henau.edu.cn; ^2^ China Animal Disease Control Center, Beijing, 102618, China, cadc.gov.cn; ^3^ National Research Center for Veterinary Medicine, Luoyang, 471000, China; ^4^ Institute of Microbiology and Epizootics, Centre for Infection Medicine, Department of Veterinary Medicine, Freie Universität Berlin, Berlin, Germany, fu-berlin.de; ^5^ Veterinary Centre of Resistance Research (TZR), Freie Universität Berlin, Berlin, Germany, fu-berlin.de; ^6^ College of Pharmaceutical Sciences, Southwest University, Chongqing, 400715, China, swu.edu.cn; ^7^ Guangdong Provincial Key Laboratory of Regional Immunity and Diseases, Department of Pathogen Biology, Shenzhen University Medical School, Shenzhen, Guangdong, China, szu.edu.cn

**Keywords:** hypervirulent *Klebsiella pneumoniae*, One Health surveillance, pathogenesis, phage therapy, virulence determinant, zoonotic transmission

## Abstract

The global spread of multidrug‐resistant hypervirulent *Klebsiella pneumoniae* (MDR‐HvKp), among which carbapenem‐resistant strains are of major concern, poses a severe threat to public health due to its high mortality rate and extremely limited treatment options. While human‐derived HvKp strains are well‐studied, animal‐origin variants remain poorly characterized. Here, we isolated a HvKp strain KPB from a swine farm in China, exhibiting high mortality and extreme virulence (LD_50_ = 20 CFU). Phylogenomic analysis of 342 *K. pneumoniae* genomes revealed that the swine‐derived KPB (sequence type 25 [ST25] lineage) clusters closely with clinical isolates, suggesting zoonotic transmission risks. Targeted mutagenesis identified *wcaJ/wzc*‐mediated capsule synthesis as the critical virulence determinant, with capsule‐deficient mutants showing 100% reduced lethality in mouse infection models. Building on this, we developed a phage therapy achieving 100% survival in infected mice at 10^1^ PFU doses. These findings highlight the evolutionary convergence of animal and human HvKp strains and propose phage‐based strategies as a promising countermeasure against infections due to HvKp. Our study underscores the urgency of One Health surveillance to mitigate zoonotic threats.

## 1. Introduction

The emergence of multidrug‐resistant hypervirulent *Klebsiella pneumoniae* (MDR‐HvKp) has become a critical global health concern due to its high mortality rates, rapid dissemination, and limited therapeutic options [1, 2]. Unlike classical *K. pneumoniae* (cKp) strains, which primarily cause opportunistic infections in immunocompromised individuals, hypervirulent strains have the ability to infect healthy hosts and cause severe, invasive diseases such as pyogenic liver abscess, meningitis, and endophthalmitis [3–5]. The convergence of antimicrobial resistance and hypervirulence in MDR‐HvKp represents a particularly alarming development, complicating clinical management and leading to poor outcomes [6–9].

Given the inability of the *Galleria mellonella* model to differentiate HvKp from cKp, mouse infection models remain the gold standard for assessing bacterial virulence in clinical research [10]. Current experimental data demonstrate a striking divergence in the median lethal doses (LD_50_): HvKp exhibits an LD_50_ ranging from 10^1^ to 10^6^ CFU, whereas cKp typically requires >10^7^ CFU to achieve comparable mortality [11]. HvKp strains are typically characterized by enhanced production of capsular polysaccharide (CPS), siderophores, such as aerobactin and salmochelin, and regulators like the products of the genes *rmpA* and *rmpA2* [12–15]. Recent genomic studies have revealed that mobile genetic elements, including virulence plasmids and integrative conjugative elements (ICEs), play critical roles in disseminating these hypervirulence traits across different *K. pneumoniae* lineages [16–19]. Despite extensive research on the epidemiology and pathogenesis of HvKp strains of human origin, data on those derived from food animals remain scarce and are only sporadically reported [20–26]. Recent evidence, however, suggests that hypervirulent sequence type 25 (ST25) *K. pneumoniae* has established sustained circulation within swine populations. Naing et al. [27] reported the emergence and long‐term persistence of ST25 HvKp in pig farms in the Netherlands from 2013 to 2020, with several human clinical isolates clustering within the same phylogenetic lineages as pig‐derived isolates, highlighting its interspecies spillover potential. Earlier outbreak investigations also documented that related ST25 lineages can cause septicemia in neonatal pigs, reinforcing swine as a potential reservoir for virulent *K. pneumoniae* [28]. While these findings collectively support the biological plausibility of zoonotic transmission, direct epidemiological evidence remains limited due to insufficient integrated animal–human surveillance.

Moreover, given the increasing failure of antibiotic therapies against MDR‐HvKp infections, alternative strategies, such as bacteriophage therapy, are being actively explored [29–34]. Phages offer several advantages, including high specificity, self‐amplification at the site of infection, and the ability to disrupt biofilms, making them promising candidates against multidrug‐resistant bacterial pathogens.

In this study, we isolated a hypervirulent *K. pneumoniae* strain, KPB, from a swine farm in China associated with exceptionally high mortality. Comparative genomics and phylogenetic analyses positioned KPB within the globally disseminated ST25 lineage, while functional studies using isogenic mutants demonstrated that CPS serves as a major determinant of virulence. Phage therapy targeting KPB was further evaluated in a mouse infection model as a potential alternative intervention. Global genomic surveillance of ST25 identified 342 isolates, revealing distinct subclades with highly conserved SNP profiles across geographically disparate regions and strong associations with human clinical disease (83.9%). Together, these observations establish ST25 as a globally disseminated, clinically relevant hypervirulent lineage and frame the importance of elucidating capsule‐mediated pathogenicity while exploring targeted interventions, such as phage therapy, to mitigate its expanding threat.

## 2. Materials and Methods

### 2.1. Bacterial Strain Isolation and Identification

In 2020, an isolate was obtained from the lung of a diseased pig on a farm in China. The utilization of the strains was approved by the Ethics Committee of Henan Agricultural University, and the consent of the sample owners was obtained. The isolate was cultured on MacConkey Inositol Adonitol Carbenicillin Agar. At this specific swine farm, 15‐day‐old piglets displayed clinical signs of illness lasting 2–5 days, with a mortality rate reaching up to 90%. Administration of oxytetracycline was ineffective in treating the condition. Postmortem analysis of two deceased piglets revealed prominent pulmonary hemorrhages, with no other significant macroscopic abnormalities observed in the remaining organs. Metagenomic sequencing of spleen and lung samples identified a predominance of *K. pneumoniae*, which was subsequently confirmed through bacterial isolation.

### 2.2. Whole Genome Sequencing and Genomic Annotation

Whole genome DNA of *K. pneumoniae* strain KPB was sequenced using the PacBio RS and Illumina MiSeq platforms (Shanghai Personal Biotechnology Co., Ltd., China). PacBio long‐read sequencing data were de novo assembled using HGAP4 (Hierarchical Genome Assembly Process 4) and CANU (v1.6), followed by iterative error correction with Illumina MiSeq short‐read data via Pilon (v1.22). Open reading frames (ORFs) were predicted and functionally annotated using the Glimmer 3.0 algorithm with a minimum coding sequence length threshold of 300 bp. Virulence genes and antimicrobial resistance genes were identified using the BIGSdb *Klebsiella* genome database and Resfinder [35, 36], respectively.

### 2.3. In Vivo Virulence Assays in Mice

To determine the LD_50_ of the antimicrobial‐resistant, capsular type K2 *K. pneumoniae* strain KPB, a murine sepsis model was established using intraperitoneal inoculation. Bacterial cultures at mid‐logarithmic phase were serially diluted in 100 μL of sterile phosphate‐buffered saline (PBS) and injected intraperitoneally into 6‐ to 8‐week‐old female BALB/c mice (*n* = 6 per group). Mice were monitored for 7 days postinfection for clinical symptoms and survival. The mice in all groups were housed under identical conditions, and the investigator assessing the various parameters was blinded to the group allocations. At predetermined time points, mice were euthanized via cervical dislocation. Following euthanasia, major organs—including the heart, liver, spleen, lungs, and kidneys—were aseptically collected and fixed in 10% neutral‐buffered formalin. Tissue samples were processed, embedded in paraffin, and stained with hematoxylin and eosin (H&E) to evaluate the extent and nature of histopathological changes.

### 2.4. In Vivo Virulence Assays in Swine

To eliminate potential interference from other pathogens, a total of 12 swine, aged 40–60 days, were tested for a range of relevant pathogens, including African swine fever virus (ASFV) antigen and antibodies, swine circovirus type 2 (PCV2) antigen, reproductive and respiratory syndrome virus (PRRSV) antigen, *Glaesserella parasuis* (GPS) antigen, *Actinobacillus pleuropneumoniae* (APP) antigen, and *Streptococcus suis* antigen. All test results were negative prior to the commencement of the experiment.

The swine were randomly assigned to two groups, each comprising six individuals. The first group was then intraperitoneally injected with 2.0 × 10^10^ CFU of KPB, and the second group was injected with PBS as a negative control. After a 14‐day observation period, animals were euthanized by intravenous injection of sodium pentobarbital (100 mg/kg body weight) via the auricular vein. Death was confirmed by the cessation of respiration and heartbeat. All procedures complied with institutional guidelines and were approved by the Animal Ethics Committee of Henan Agricultural University.

Following euthanasia, a comprehensive necropsy was performed. The heart, liver, spleen, lungs, kidneys, brain, and multiple lymph nodes were collected for gross pathological examination and histological assessment. Tissue specimens were fixed in 10% neutral‐buffered formalin, embedded in paraffin, sectioned, and stained with H&E to evaluate the presence and extent of pathological lesions.

### 2.5. Plasmid Elimination Assay

Plasmid elimination was performed following established protocols with minor modifications [37]. Single colonies were inoculated into fresh LB broth and incubated at 37 °C for 12 h. A 300 µL aliquot of the bacterial culture was then transferred into 30 mL of fresh LB broth containing 0.3% SDS and incubated overnight at 45 °C. The cultures were subsequently plated onto nonselective LB agar plates. To verify the presence of the virulence plasmid, polymerase chain reaction (PCR) targeting the *iucA* gene was performed on 100 randomly selected colonies from each treatment group. The plasmid loss frequency was calculated by dividing the total number of colonies tested (*n* = 100) by the number of plasmid‐free colonies.

### 2.6. Conjugation Assay

Conjugation experiments were performed using rifampin‐resistant *Escherichia coli* EC600 or hygromycin‐resistant *K. pneumoniae* yz6 as recipients. Donor (KPB) and recipient strains were cultured to logarithmic phase in LB broth at 37°C. Donor (100 µL) and recipient (400 µL) cells were mixed and spotted onto LB agar, incubated overnight at 37 °C, and then harvested for plating on selective media. Transconjugants were selected on LB agar containing tetracycline (8 mg/L) and rifampin (128 mg/L) for EC600 or tetracycline (16 mg/L) and hygromycin B (128 mg/L) for yz6. MICs were determined by broth microdilution following CLSI guidelines, with *E. coli* ATCC 25922 as the quality control.

### 2.7. Plasmid Stability in Transconjugants

A single colony was isolated from an LB agar plate and inoculated into LB broth. Serial subcultures of each purified transconjugant were performed over a 2‐week period, with 10 µL of the bacterial suspension transferred into 10 mL of fresh LB broth every 12 h. Plasmid stability in the transconjugants was evaluated by streaking each subculture onto fresh LB agar plates. Three individual colonies were randomly selected from each subculture for antibiotic resistance profiling and PCR detection of the *iucA* gene, which is associated with the virulence plasmid.

### 2.8. Virulence Gene Knockout Assay

Knockout of the *rmpA*, *iroB*, *wcaJ*, *wzc*, and *peg-344* genes in *K. pneumoniae* KPB was performed using the clustered regularly interspaced short palindromic repeats (CRISPR) system [38]. Genome editing was conducted according to the two‐plasmid system protocol, pCasKP‐pSGKP, with a 20‐bp spacer designed using the sgRNAcas9 software [39]. The spacer sequence was subsequently ligated into the pSGKP‐km plasmid. The two plasmids, pCasKP‐apr and pSGKP‐km, were electroporated sequentially into the target strain to cleave the virulence genes. The primers used in this procedure are listed in Supporting Information 1: Table S1 for reference. Antibiotics were added at the following concentrations: 50 mg/L apramycin and 50 mg/L kanamycin for *K. pneumoniae* isolates.

### 2.9. Mucoviscosity Assay

Overnight LB cultures were adjusted to OD_600_ = 1.0 with sterile LB, and initial OD_600_ was measured. After centrifugation at 1000 × g for 5 min, with the supernatant OD reflecting mucoviscosity levels.

### 2.10. Capsule Extraction and Quantification

Uronic acid quantification was performed as previously described with minor modifications [40]. Overnight bacterial cultures grown in LB broth were diluted into fresh LB to an initial OD_600_ of 0.2 and incubated at 37 °C for 4 h with shaking. A 500 μL aliquot of the resulting culture was combined with 100 μL of 1% ZWITTERGENT prepared in 100 mM citric acid and incubated at 50 °C for 20 min to extract CPS. The mixture was centrifuged at maximum speed for 5 min at room temperature to remove cellular debris. Then, 300 μL of the clarified supernatant was transferred into 1.2 mL of cold absolute ethanol, followed by incubation at 4 °C for 20 min to precipitate the capsular material. After centrifugation at high speed for 5 min, the resulting pellet was air‐dried and resuspended in 200 μL of distilled water. To initiate the colorimetric reaction, 1.2 mL of freshly prepared 12.5 mM sodium tetraborate in concentrated sulfuric acid was added to each sample. The tubes were vortexed and heated at 100 °C for 5 min and then rapidly cooled on ice for 10 min. Subsequently, 20 μL of 0.15% 3‐hydroxydiphenyl dissolved in 0.5% NaOH was added. After thorough mixing, absorbance was measured at 520 nm using a spectrophotometer. Glucuronic acid concentrations were quantified based on a standard curve and normalized to the culture optical density (OD_600_), with results expressed as micrograms per OD unit. Additionally, CPS fractions extracted from equal volumes of *K. pneumoniae* cultures were separated through 8% SDS‐PAGE, with subsequent Alcian blue staining, and then subjected to visualization using ImageJ software.

### 2.11. Cell Line Preparation

HEp‐2 human epithelial cells and RAW264.7 murine macrophages used in this study were obtained from the laboratory’s established cell line repository. Both cell lines were originally purchased from the Cell Bank of the Shanghai Institute of Cell Biology, Chinese Academy of Sciences (Shanghai, China) and have been routinely authenticated and verified to be free of mycoplasma contamination. HEp‐2 cells were selected because they are widely used as a reproducible in vitro epithelial adhesion model for evaluating the attachment capacity of *K. pneumoniae* and other respiratory or opportunistic pathogens. HEp‐2 cells were maintained in DMEM supplemented with 10% fetal bovine serum (FBS), while RAW264.7 cells were cultured in DMEM containing 10% FBS under standard incubation conditions (37 °C and 5% CO_2_). All cell‐based experiments were conducted in accordance with the guidelines of Henan Agricultural University and were approved by the Animal Care Committee of Henan Agricultural University.

### 2.12. Adhesion Assay

The adhesion ability was evaluated using HEp‐2 epithelial cells. Cells were seeded in 24‐well plates and cultured overnight. Bacterial strains were grown to mid‐log phase, washed, and resuspended in DMEM without antibiotics. HEp‐2 cells were infected at a multiplicity of infection (MOI) of 100 and incubated for 1.5 h at 37 °C with 5% CO_2_. After incubation, wells were washed three times with PBS to remove nonadherent bacteria, and cells were lysed with 0.1% Triton X‐100. The number of adherent bacteria was quantified by plating serial dilutions on LB agar to determine colony‐forming units (CFUs).

### 2.13. Phagocytosis Assay

The phagocytic uptake of *K. pneumoniae* strains by RAW264.7 macrophages was assessed using a gentamicin protection assay. RAW264.7 cells were seeded in 24‐well plates and incubated overnight. Bacterial cultures were prepared as described above and added at an MOI of 10. After 90 min of infection, cells were washed and incubated with medium containing 100 mg/L gentamicin for 1 h to kill extracellular bacteria. Cells were then washed and lysed, and internalized bacteria were enumerated by plating on LB agar. Data are expressed as log CFU/mL.

### 2.14. Phylogenetic Analysis and Visualization

A maximum likelihood phylogenetic tree was constructed based on core‐genome single‐nucleotide polymorphisms (SNPs) derived from whole‐genome sequences of the *K. pneumoniae* isolates. Genomic sequences were aligned using Roary v3.13.0 [41], and SNPs were extracted for tree construction. The phylogenetic tree was inferred using IQ‐TREE v2.2.2.2 (MFP model, bootstrap = 1000) [42], clade definition: FigTree v1.4.4 (http://tree.bio.ed.ac.uk/software/figtree/), and visualization with bootstrap ≥ 70% as stable branches. The resulting tree was visualized and annotated using chiplot (https://www.chiplot.online/tvbot.html), with concentric rings denoting the presence of virulence genes (*iucA*, *rmpA*, *iroB*, and *rmpA2*). Clinical isolates were marked with red dots, and the focal strain (KPB) was highlighted with a red branch label.

### 2.15. Global Distribution Mapping

Publicly available genome metadata were retrieved from the NCBI database and relevant published datasets. Geographic origin was determined based on sample metadata, and isolate counts per country were aggregated. A world map indicating country‐level distribution and frequency of reported strains was generated using Tableau (version 2025.1). Countries were shaded according to isolate count, with darker colors indicating higher prevalence.

### 2.16. Phage Therapy

To evaluate the in vivo therapeutic efficacy of the phage, 4 groups of 6‐ to 8‐week‐old female BALB/c mice (*n* = 6 per group) were obtained from Liaoning Changsheng Biotechnology Co., Ltd. Mice of groups 1 and 2 were infected with strain KPB at a dose of 2.0 × 10^4^ CFU in a volume of 200 μL. In the phage treatment group (group 1), mice were intraperitoneally injected with 200 μL of the phage solution at a dose of 2.0 × 10^1^ PFU, 2 h postinfection. In the control group (group 2), mice were intraperitoneally injected with 200 μL PBS. A third group of mice was included in the experiment, in which the animals received an intraperitoneal injection of 200 μL of PBS but were not infected with strain KPB.

All three groups were monitored for 7 days before euthanasia. For histopathological analysis, tissues from the heart, liver, spleen, lungs, and kidneys were collected, fixed in 4% paraformaldehyde, dehydrated, embedded in paraffin, and stained with H&E.

### 2.17. Statistical Analysis

Data were analyzed using two‐tailed Student’s *t*‐tests in GraphPad Prism (GraphPad Software Inc., San Diego, CA, USA), and results were presented as mean ± standard deviation (SD). Statistical significance between groups was determined using the *t*‐test, with *p*‐values less than 0.05 considered significant.

## 3. Results

### 3.1. Characterization of the HvKp Strain KPB

The KPB strain used in this study belongs to ST25 and serotype K2. Genomic analysis revealed that this strain harbors a chromosome of 5,342,761 bp and three plasmids of 252,792 bp, 40,449 bp, and 4092 bp in size. The largest plasmid, designated pKPB_Vir, carries the *iucABCD-iutA* operon (Figure 1A). Moreover, this plasmid also carries several antimicrobial resistance genes, including *qnrS1*, *bla*_LAP−2_, *tet*(A), *aph*(3″)‐Ib, *aph*(6)‐Id, and *sul2* as well as a copper resistance gene cluster comprising the genes *copABDR*. To further characterize the virulence‐associated features of pKPB_Vir, we conducted a comparative plasmid analysis using BRIG against the canonical virulence plasmid of the hypervirulent strain NTUH‐K2044. This alignment revealed that pKPB_Vir lacks the *rmpA2* gene (Supporting Information 2: Figure S1)—a noteworthy distinction given the well‐recognized contribution of *rmpA2* to enhanced hypermucoviscosity and hypervirulence in classical HvKp plasmids. Consistent with this interpretation, several key chromosomal virulence‐associated genes were identified, including *wcaJ*, *wzc*, *rmpA*, *peg-344*, and *iroB*, supporting the notion that the hypervirulence of KPB is shaped by a combination of plasmid‐borne and chromosomally encoded determinants.

Figure 1Characterization of the virulence plasmid and phenotypic assessment of the KPB strain. (A) Circular map of the virulence plasmid (pKPB_Vir, 252,792 bp) carried by the KPB strain. Arrows represent annotated genes, with red indicating antimicrobial resistance genes and blue indicating virulence‐associated genes. (B) Plasmid stability assay of the pKPB_Vir conjugative plasmid in recipient strains KY1 and KE1 over 30 days under nonselective conditions. The percentage of colonies retaining the plasmid was determined. (C–H) LD_50_ determination of KPB, KPB_Δ*peg-344*, KPB_Δ*wcaJ*/Δ*wzc*, KPB_Δ*rmpA*, KPB_Δ*iucABCD-iutA*, and KPB_Δ*iroB* in a mouse intraperitoneal infection model, respectively. Female BALB/c mice (*n* = 6 per group) were challenged with serial dilutions of KPB, and survival was monitored over 7 days.

**Figure:**
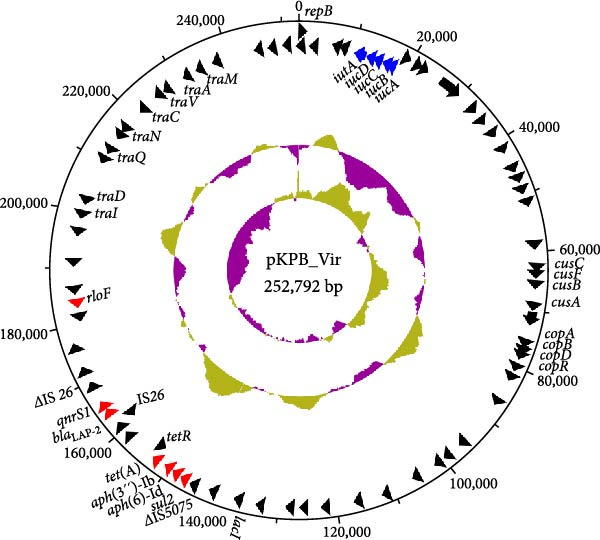
(A)

**Figure:**
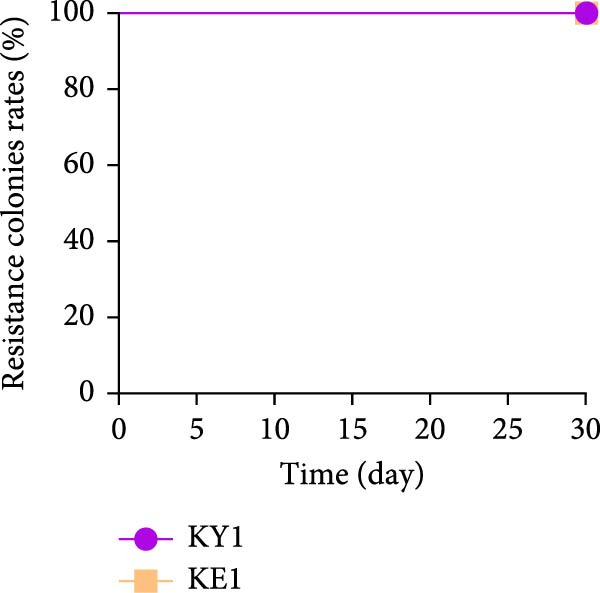
(B)

**Figure:**
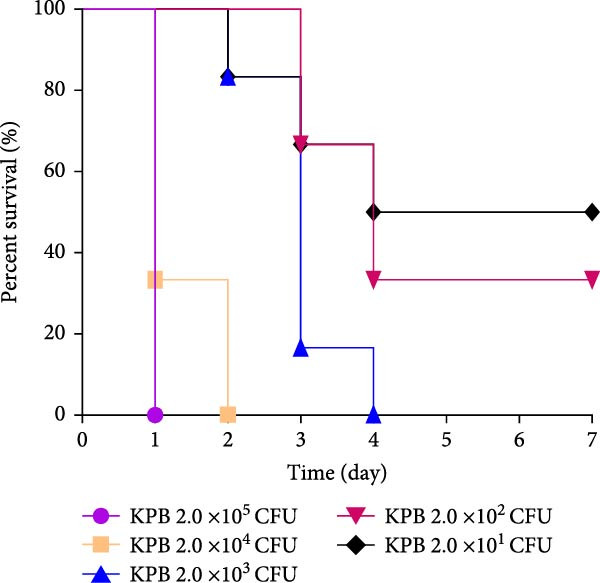
(C)

**Figure:**
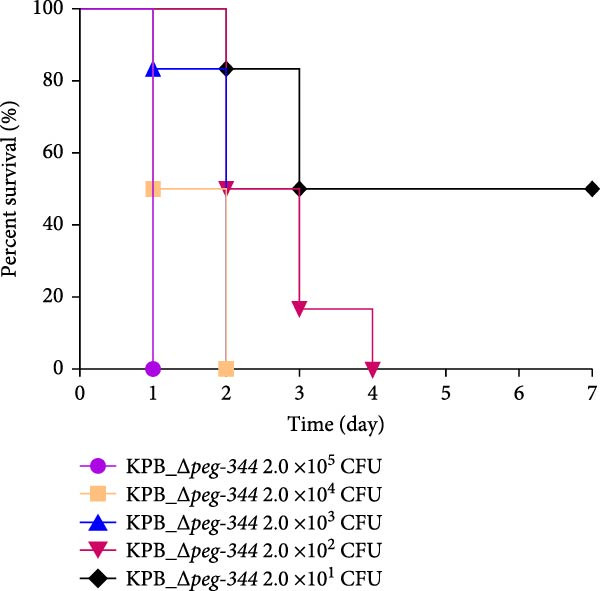
(D)

**Figure:**
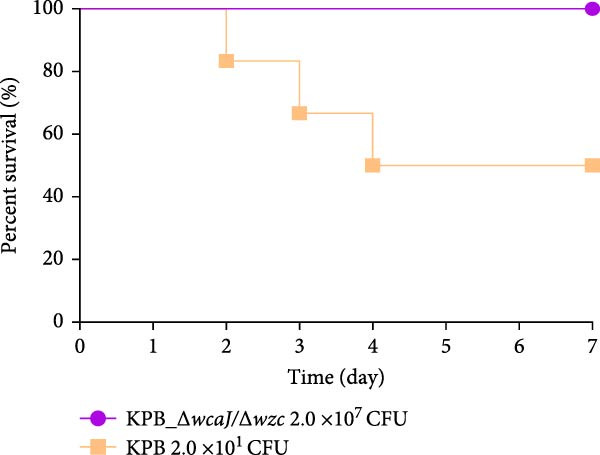
(E)

**Figure:**
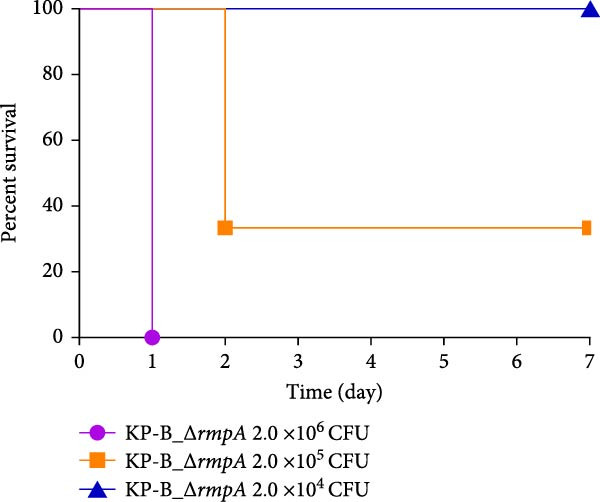
(F)

**Figure:**
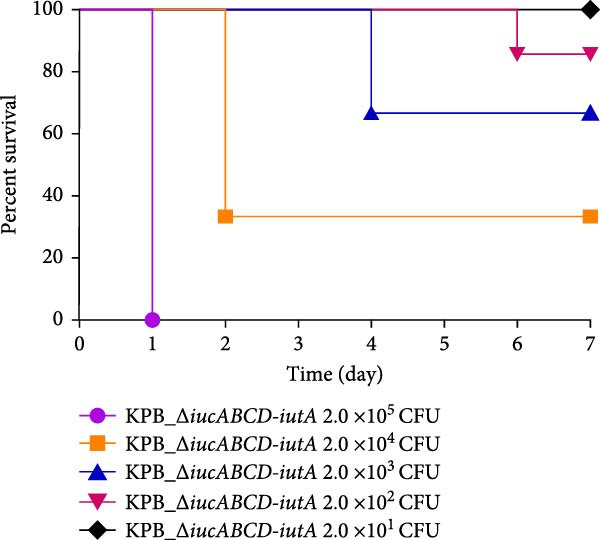
(G)

**Figure:**
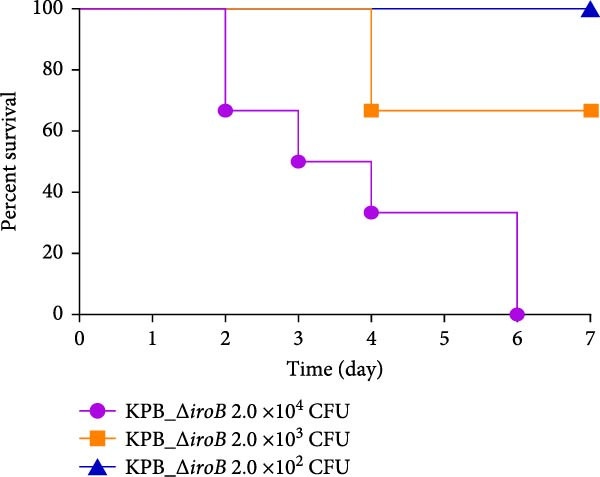
(H)

### 3.2. Global Dissemination and Genomic Architecture of the *K. pneumoniae* ST25 Lineage

To investigate the global epidemiology and genomic characteristics of the *K. pneumoniae* ST25 clone, we screened 22,509 publicly available *K. pneumoniae* genomes deposited in NCBI between 2005 and 2024. Among them, a total of 342 ST25 isolates were identified through multilocus sequence typing (MLST) and subjected to core‐genome SNP (cgSNP) phylogenetic analysis. The resulting phylogeny revealed distinct subclades within ST25, with branch lengths indicative of genomic divergence across strains (Figure 2A, Supporting Information 1: Table S2). To strengthen lineage comparisons, we expanded our dataset to include 291 genomes representing ST25, ST11, and ST23 (Supporting Information 2: Figure S2 and Supporting Information 1: Table S3). The revised core‐genome phylogeny confirmed that ST25, ST11, and ST23 form distinct and independently evolved clades. Within ST25, the swine‐derived KPB isolate clustered tightly with human clinical strains, highlighting close phylogenetic relatedness and suggesting that this lineage may circulate across host species. This cross‐host clustering provides context for interpreting the observed geographic distribution and potential zoonotic transmission of ST25. Remarkably, isolates from geographically disparate regions—including Japan, China, and Norway—exhibited highly conserved SNP profiles, consistent with clonal expansion and global dissemination of this hypervirulent lineage.

Figure 2Phylogenetic relationships and global distribution of 342 ST25 *K. pneumoniae* isolates. (A) Maximum‐likelihood phylogenetic tree based on the core genome alignment of 342 *K. pneumoniae* isolates. Presence of virulence‐associated genes is indicated in concentric colored bands: *iucA* (aerobactin synthesis, pink), *rmpA* (mucoid phenotype regulator, yellow), *iroB* (salmochelin synthesis, blue), and *rmpA2* (mucoid phenotype regulator, green). Isolates from clinical sources are marked with red dots, while others are marked with black dots. The red branch indicates the position of the KPB strain. (B) Geographic distribution of the 342 ST25 *K. pneumoniae* isolates across 23 countries/regions. The number of isolates per country is represented by the intensity of red shading, with darker colors indicating higher isolate counts. China and Japan contribute the most strains (43 and 67, respectively), followed by Norway (37), the United Kingdom (31), and the United States (26).

**Figure:**
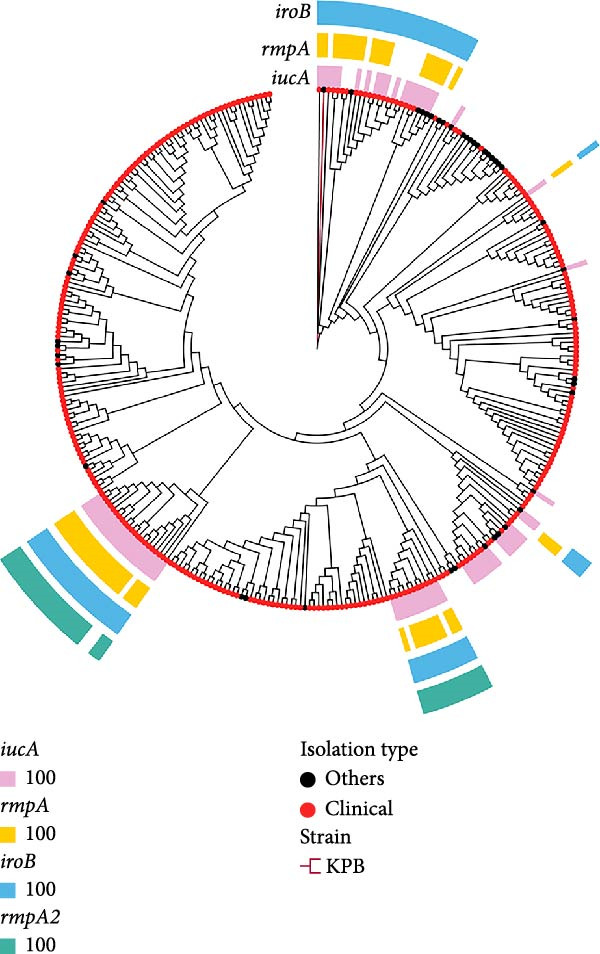
(A)

**Figure:**
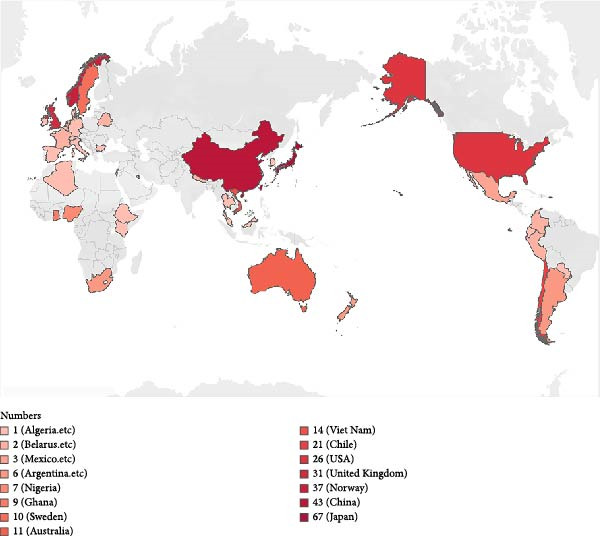
(B)

Mapping the global distribution of ST25 isolates revealed Japan as the dominant reservoir (*n* = 67, 19.6%), followed by China (*n* = 43, 12.6%), Norway (*n* = 37, 10.8%), the United Kingdom (*n* = 31, 9.1%), the United States (*n* = 26, 7.6%), and Chile (*n* = 21, 6.1%) (Figure 2B). Among these ST25 strains, 83.9% (*n* = 287) originated from human clinical specimens, underscoring their strong association with human disease. The remaining isolates were primarily derived from livestock and healthcare environments, implicating both zoonotic transmission and nosocomial spread in the ongoing expansion of this clone.

Phylogenetic annotation of virulence determinants revealed subclade‐specific distributions of hypervirulence loci. Notably, *rmpA*, a key regulator of the hypermucoid phenotype, was consistently detected among isolates from East Asia—particularly Japan and China—where ST25 was also most prevalent. Other virulence‐associated loci, such as *iroB* and *iucABCD-iutA*, exhibited cosegregation with distinct phylogenetic clusters, suggesting the parallel acquisition or maintenance of virulence plasmids during clonal evolution. Collectively, these findings establish ST25 as a globally disseminated and clinically adapted hypervirulent *K. pneumoniae* lineage with evidence of zoonotic and nosocomial transmission potential.

### 3.3. Comparative Genomic Features of ST25 Versus Other High‐Risk *K. pneumoniae* Lineages

ST25 exhibited a hybrid pathogenic profile that distinguishes it from other prevalent *K. pneumoniae* lineages (Table 1). Similar to classical hypervirulent clones such as ST23 and ST65, ST25 carried key virulence determinants including *rmpA*, *iuc*, *iro*, *ybt*, and *peg-344*, indicating its capacity to express a hypervirulent phenotype. However, unlike these predominantly community‐associated HvKp lineages, ST25 was detected across both community and hospital settings and occurred in multiple hosts, including humans and swine, supporting a potential cross‐species transmission risk. Compared with MDR high‐risk clones such as ST11, ST15, and ST258, ST25 harbored a more diverse plasmid background (IncFII, IncFIB/K, IncHI2, IncN, and IncX3) and frequently encoded carbapenemases (*bla*_KPC_, *bla*_NDM_, and *bla*_OXA_), highlighting its notable role at the interface of hypervirulence and multidrug resistance.

**Table 1:** Comparative analysis of virulence determinants, plasmid content, and antimicrobial resistance profiles among hypervirulent *Klebsiella pneumoniae* ST25 and other high‐risk lineages [27, 43–47].

Sequence type (ST)	Geographic distribution	Infection types	Major KL types and virulence determinants	Typical plasmid replicon types	Common resistance genes
ST11	Global	Nosocomial outbreaks (pneumonia and bacteremia)	KL64, KL47, KL2, and KL1 *iuc*, *rmpA*, and *iro*	IncFII, IncHI1B/IncFIB, and IncR	*bla*_KPC_, *bla*_NDM_, *bla*_CTX-M_, *bla*_SHV_, *mcr*, *tet*(A), and *tmexCD1-toprJ1*
ST15	Europe, Asia, and Africa	Nosocomial/community (urinary tract infection (UTI), bloodstream, and respiratory)	KL112 and KL19 *rmpA2*, *iuc*, and *ybt*	IncFII, IncHI1B/IncFIB, and ColKP3	*bla*_CTX-M_, *bla*_SHV_, *bla*_OXA_, and *fosA*
ST23	East Asia, Europe, and North America	Hypervirulent infections: liver abscess, bloodstream infection (BSI), and meningitis (especially in healthy individuals) Healthcare‐associated infections (HAI): pneumonia and UTI	KL1, KL2, and KL10 *rmpA*, *rmpA2*, *iuc*, *iro*, *ybt*, and *peg-344*	IncFII, IncHI2, and IncN	*bla*_KPC_, *bla*_NDM_, *bla*_CTX-M_, *bla*_SHV_, *mcr*, *tet*(A), *aac*(*6′*)*-Ib-cr*, and *OqxA*/*OqxB*
ST25	Global	Community and hospital; animal–human link suspected	KL2 *rmpA*, *iuc*, *iro*, *ybt*, and *peg-344*	IncFII, IncFIB/K, IncHI2, IncN, and IncX3	*bla*_KPC_, *bla*_NDM_, *bla*_SHV_, *bla*_OXA_, *aac*(*6′*)*-Ib-cr*, *aadA2*, *fosA*, and *OqxA*/*OqxB*
ST65	East Asia and Southeast Asia	Community‐acquired infections (CAI): liver abscess and pyogenic meningitis	KL2 and KL14 *iuc*, *iro*, *rmpA*/*rmpA2*, and *ybt*	IncFII, IncHI2, IncN, and IncR	*bla*_KPC_, *bla*_NDM_, *bla*_CTX-M_, *bla*_SHV_, *mcr*, and *tet*(A)
ST86	East Asia and Europe	Community‐acquired invasive infections: liver abscess and BSI	KL2, KL10, and KL64 *rmpA*/*rmpA2* and *iuc*/*iro*	IncFII, IncHI2, IncN, and IncR	*bla*_KPC_, *bla*_NDM_, *bla*_CTX-M_, *bla*_SHV_, *mcr*, and *tet*(A)
ST258	Global	Nosocomial outbreaks (bloodstream and respiratory)	KL106, KL107, and KL64 *rmpA*/*rmpA2*, *iuc*, and *ybt*	IncFII and IncHI1B/IncFIB	*bla*_KPC_, *bla*_VIM_, *bla*_NDM_, and *bla*_OXA_

### 3.4. Conjugative Transfer of Plasmid pKPB_Vir to *K. pneumoniae* and *E. coli* Recipients

To investigate the horizontal transmission potential of a plasmid pKPB_Vir, we performed biparental conjugation assays using rifampicin‐resistant *E. coli* EC600 and hygromycin‐resistant *K. pneumoniae* yz6. Selection on antibiotic‐containing media confirmed the successful acquisition of the virulence plasmid by both recipient strains. PCR detection of key plasmid‐borne virulence genes in transconjugants, along with phenotypic assays, validated the presence and functional expression of the respective plasmid genes. These findings demonstrated that the plasmid pKPB_Vir is capable of efficient horizontal transfer not only within *K. pneumoniae* populations but also across species boundaries to *E. coli*, thereby underscoring the risk of cross‐species dissemination of virulence traits.

### 3.5. Stability of the Virulence Plasmid in Transconjugants During Serial Passage

To assess the stability of the transferred plasmid, we passaged the *K. pneumoniae* and *E. coli* transconjugants daily for 30 consecutive days under nonselective conditions. At regular intervals, plasmid presence was evaluated via plasmid extraction and PCR targeting virulence‐associated genes. Both transconjugant strains retained the plasmid throughout the 30‐day period, with no detectable loss or structural rearrangements (Figure 1B). This result highlights the strong stability of plasmid pKPB_Vir in different bacterial hosts, suggesting it can persist even in the absence of a selective pressure and may contribute to the long‐term maintenance and spread of virulence within microbial communities.

### 3.6. Construction of Isogenic Mutant Derivatives of the HvKp Isolate KPB

Isogenic mutant derivatives of the HvKp strain KPB were successfully generated (Supporting Information 1: Table S1). Whole genome sequencing confirmed that all mutant strains were isogenic and harbored the desired genetic modifications, which were verified by comparison with the genome of the wild‐type parental strain.

### 3.7. Virulence Determinant Hierarchy in KPB Pathogenesis

In a mouse intraperitoneal infection model using BALB/c female mice, we evaluated the lethality of *K. pneumoniae* strain KPB and its mutants lacking key virulence factors. Strain KPB demonstrated an exceptionally low LD_50_ of 20 CFU (Figure 1C), underscoring the strain’s hypervirulent phenotype. However, deletion of *peg-344* did not attenuate virulence, as the mutant strain displayed virulence levels comparable to the wild‐type strain KPB (Figure 1D). The *wcaJ*/*wzc* deletion mutant exhibited 100% survival at 10^7^ CFU (Figure 1E), indicating its attenuation to the virulence level of cKp. The *rmpA* mutant showed complete survival (100%) at doses ≤10^4^ CFU (Figure 1F). In contrast, mutants lacking the siderophore‐related *iucABCD-iutA* locus achieved >80% survival at 10^2^ CFU (Figure 1G), while the *iroB* mutant demonstrated 100% survival at the same dose (Figure 1H). Notably, the attenuated virulence of siderophore‐deficient strains was significantly less pronounced than that of capsule‐deficient mutants, highlighting the predominant contribution of the CPS to KPB‐based lethality.

### 3.8. Systemic Multiorgan Pathology in Swine Following Infection With KPB

To characterize the pathological consequences of an HvKp infection in a large animal model, we conducted gross and histopathological examinations in swine challenged with KPB.

Gross pathological evaluation revealed widespread and severe systemic involvement. Both thoracic and abdominal cavities contained abundant fibrinous exudates and turbid, yellow, serous effusions, indicative of acute inflammation. The lungs exhibited multifocal hemorrhagic consolidation with pronounced red discoloration, while the kidneys displayed scattered pale necrotic lesions on the cortical surface. Notably, lymph nodes were markedly enlarged and diffusely hemorrhagic, suggestive of vascular disruption and systemic immune activation.

**Figure 3:**
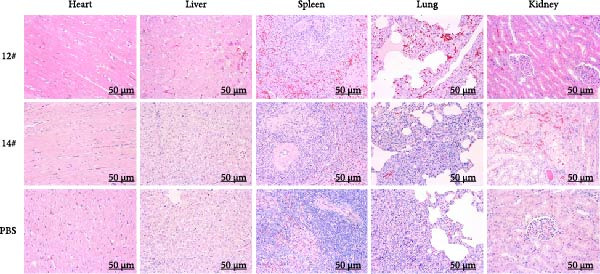
H&E staining in swine organs following KPB challenge. Representative H&E stained sections of heart, liver, spleen, lung, and kidney tissues from swine infected with strain KPB (12# and14#) and PBS–treated controls. (Scale bars, 50 μm).

Histopathological analysis further confirmed organ‐specific pathological features associated with the HvKp infection (Figure 3). Pulmonary tissues showed interstitial pneumonia characterized by alveolar septal thickening, dense neutrophilic infiltrates, and intra‐alveolar accumulation of proteinaceous exudates. Hemorrhagic lymphadenitis was observed in lymph nodes, accompanied by cortical architecture effacement and extensive erythrocyte extravasation. Renal sections revealed interstitial nephritis with evidence of tubular epithelial degeneration and perivascular leukocytic infiltration. The spleen exhibited marked capsular thickening and red pulp expansion, while hepatic tissue showed dilated sinusoids and Kupffer cell hyperplasia. Widespread vascular congestion and autolytic changes were consistently observed across all examined organs, consistent with fulminant septic progression.

These findings collectively demonstrated that HvKp induces rapid‐onset and disseminated multiorgan pathology in swine, reinforcing its pathogenic potential in large mammalian hosts and highlighting the utility of this model for evaluating systemic infection outcomes.

### 3.9. Characterization of CPS Production in Wild‐Type and Knockout Strains

To dissect the role of specific loci in *K. pneumoniae* capsule biogenesis, we quantified mucoviscosity, uronic acid levels, and ultrastructural features in wild‐type KPB and isogenic mutants (∆*wcaJ*, ∆*wzc*, ∆*rmpA*, ∆*iroB*, ∆*iucABCD-iutA*, and ∆*peg-344*).

In the mucoviscosity assay (Figure 4A), the wild‐type strain (KPB) exhibited significantly higher mucoviscosity (OD_600_) compared to the knockout strains, KPB ∆*wcaJ*/∆*wzc* and KPB ∆*rmpA* (*p* < 0.0001). However, mucoviscosity assays revealed no statistically significant differences between the wild‐type KPB strain and the ∆*iroB*, ∆*peg-344*, or ∆*iucABCD-iutA* mutants.

Figure 4Capsule production, adherence, phagocytosis, and ultrastructural characterization of *K. pneumoniae* mutants. (A, B) Mucoviscosity (OD_600_) and uronic acid production (µg/OD_600_) of the wild‐type KPB strain and its isogenic mutants, including Δ*wcaJ*, Δ*wzc*, Δ*rmpA*, Δ*iroB*, Δ*iucABCD-iutA*, and Δ*peg-344*, were quantified. (C, D) Bacterial adhesion to HEp‐2 cells and phagocytosis by RAW264.7 macrophages were evaluated. Adhesion was quantified as CFUs after 1.5 h incubation with HEp‐2 cells, while intracellular survival was measured 2 h postphagocytosis in RAW264.7 cells. (E) Transmission electron microscopy (TEM) images of KPB (wild‐type), ∆*wcaJ*, ∆*wzc*, and ∆*rmpA* strains (scale bar: 500 nm). Statistical significance of the differences between the WT control group and the mutant groups was analyzed by unpaired *t*‐tests, two‐tailed ( ^∗∗∗^ *p*  < 0.001,  ^∗∗∗∗^ *p*  < 0.0001, ns, not significant). (F) Alcian blue staining of capsular polysaccharides in wild‐type KPB and capsule‐deficient mutants. Total acidic CPS extracted from each strain was separated by 8% SDS‐PAGE and visualized by Alcian blue staining.

**Figure:**
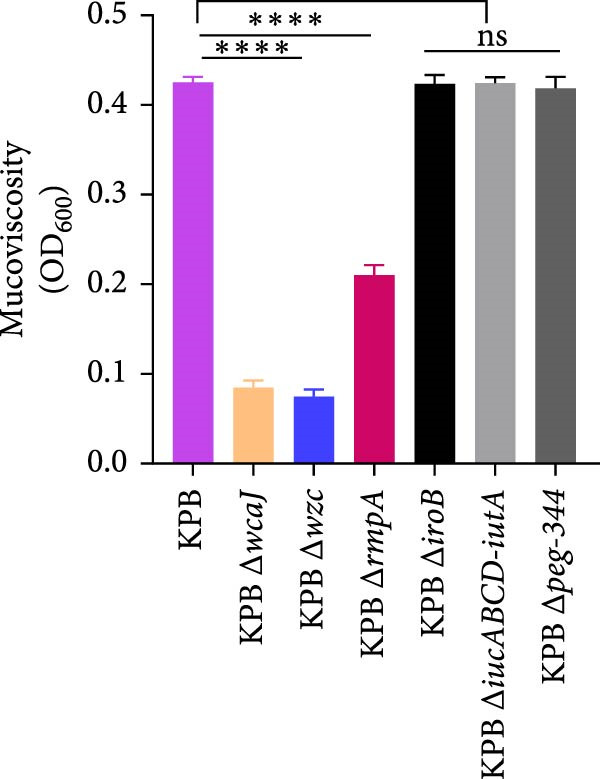
(A)

**Figure:**
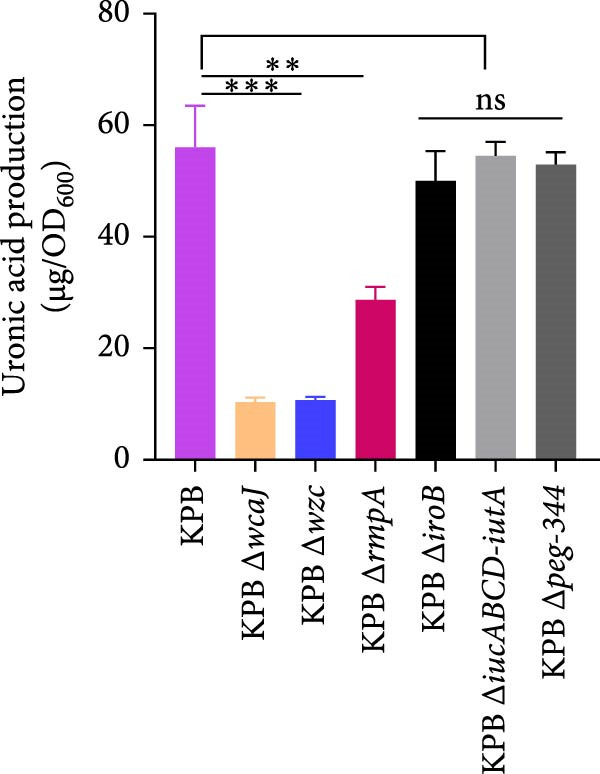
(B)

**Figure:**
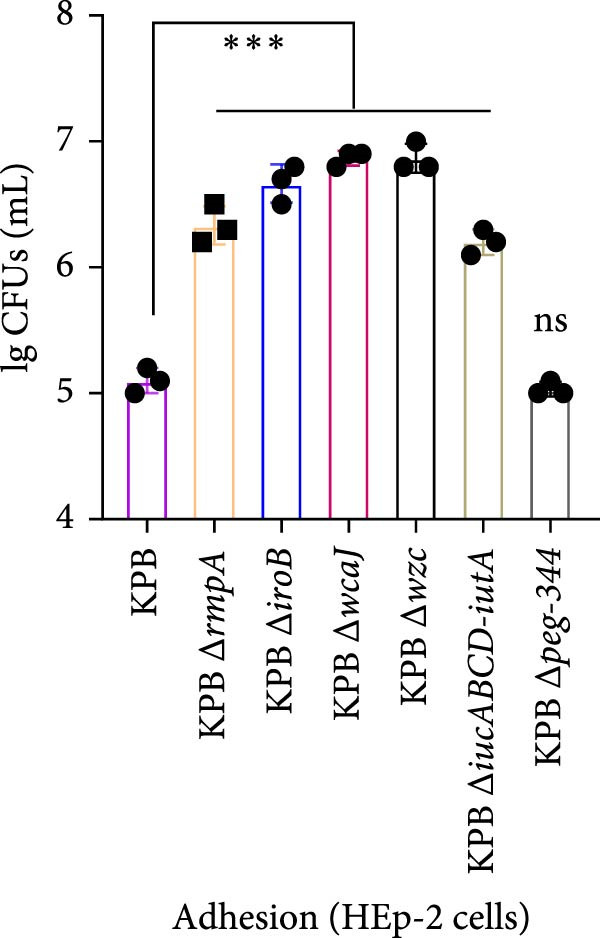
(C)

**Figure:**
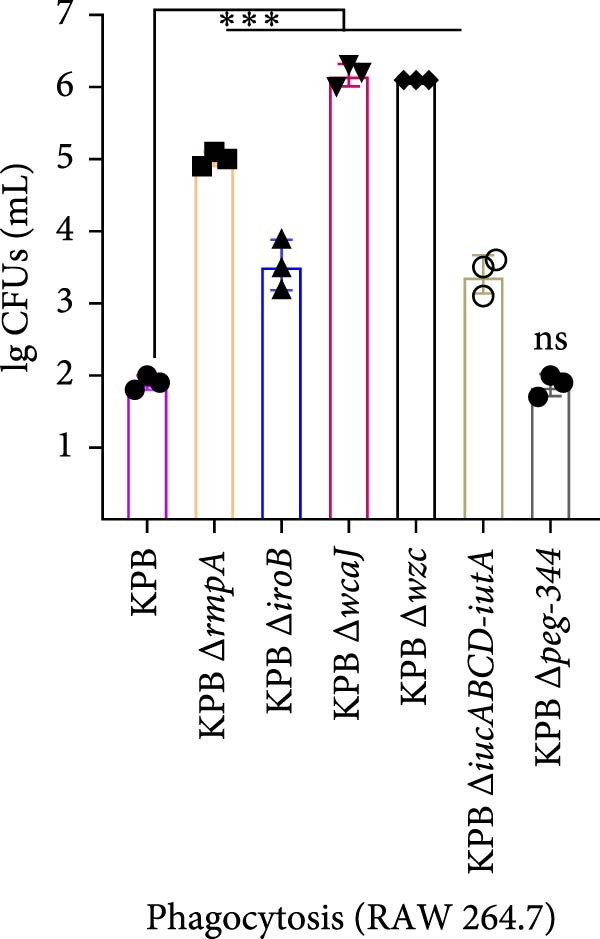
(D)

**Figure:**
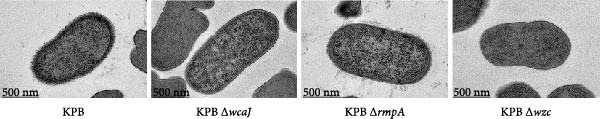
(E)

**Figure:**
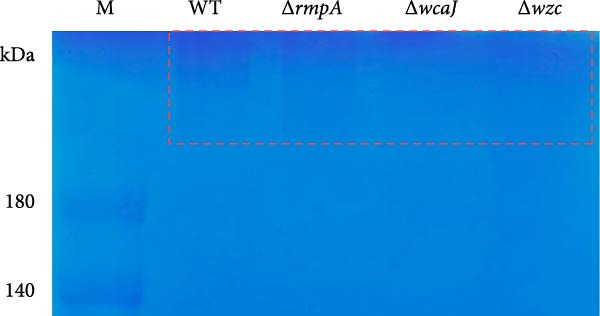
(F)

Similarly, uronic acid quantification revealed significantly reduced yields in the ∆*wcaJ*/∆*wzc* (*p*  < 0.001) and ∆*rmpA* (*p*  < 0.01) mutants compared to the wild‐type strain, whereas the ∆*iroB*, ∆*peg-344*, and ∆*iucABCD-iutA* mutants showed no statistically significant differences (Figure 4B). These findings underscore the pivotal role of *wcaJ*/*wzc* in CPS biosynthesis and highlight *rmpA* as a critical regulatory node in this pathway.

To visualize the impact of the deletions on capsule thickness, transmission electron microscopy (TEM) was employed (Figure 4E). The images revealed that the wild‐type strain KPB had a robust capsule, while both knockout strains KPB ∆*wcaJ*/∆*wzc* and KPB ∆*rmpA* showed a marked decrease in capsule thickness, corroborating the results from the mucoviscosity and uronic acid production assays. This suggests that the genes targeted in the knockout strains are fundamental to maintaining capsular integrity and polysaccharide production.

To further validate CPS abundance, Alcian blue staining was performed to detect total acidic polysaccharides (Figure 4F). In agreement with the biochemical assays, the wild‐type KPB strain and ∆*rmpA* mutant displayed a visibly stronger Alcian blue–positive CPS band compared with the ∆*wcaJ* and ∆*wzc* strains, which showed markedly diminished staining intensity. These results confirm substantial reductions in CPS production in capsule‐deficient mutants and reinforce the essential contributions of *wcaJ*/*wzc* to capsule biosynthesis and *rmpA* to its transcriptional regulation.

### 3.10. Adhesion and Phagocytosis Analysis of KPB and Knockout Strains

To assess the contribution of virulence‐associated genes to host interaction, we examined bacterial adhesion to HEp‐2 epithelial cells and phagocytic uptake by RAW264.7 macrophages. Deletion of *wcaJ*, *wzc*, *rmpA*, *iroB*, and *iucABCD-iutA* significantly impaired adhesion to HEp‐2 cells compared to the wild‐type KPB strain, while the *peg-344* mutant showed no significant change (Figure 4C). Loss of *wcaJ*/*wzc* markedly increased susceptibility to phagocytosis, as did deletion of *rmpA*, while Δ*peg-344* exhibited similar intracellular survival levels to the wild‐type strain (Figure 4D). These results suggest that *peg-344* is dispensable for bacterial adhesion and resistance to phagocytosis, whereas *rmpA* and *wcaJ*/*wzc* play more critical roles in mediating host‐pathogen interactions.

### 3.11. Characterization of the Phage vB_KpnP_B1

The bacteriophage vB_KpnP_B1 was classified within the order Caudovirales and the family Podoviridae. TEM revealed an icosahedral capsid ~55 nm in diameter and a short tail, consistent with typical podophage morphology. Host‐range assessment demonstrated a narrow lytic spectrum restricted to *K. pneumoniae* strains of the K2 serotype. Whole‐genome sequencing further showed that vB_KpnP_B1 possesses a 44,211 bp double‐stranded DNA genome lacking identifiable lysogeny‐related or virulence‐associated genes.

To determine whether phage therapy led to the emergence of phage‐resistant mutants, surviving mice were euthanized posttreatment, and their blood, liver, lung, and spleen were homogenized for bacterial enumeration. No bacteria were recovered from any tissue, demonstrating effective in vivo clearance by the therapeutic phage, which further confirms the absence of phage‐resistant bacterial populations and underscores the reliability of vB_KpnP_B1 in targeted bacterial elimination.

### 3.12. Phage Therapy Confers Complete Protection Against KPB Infection in a Mouse Model

To assess the therapeutic potential of bacteriophage treatment against KPB, we employed a lethal mouse infection model (Figure 5A). Phage vB_KpnP_B1 (2.0 × 10^1^ PFU) was administered 2‐h postinfection to mice intraperitoneally challenged with 2.0 × 10^4^ CFU of wild‐type KPB. The phage‐treated mice exhibited 100% survival over a 7‐day observation period (Figure 5B), whereas all positive control animals receiving KPB + PBS succumbed within 24 h, consistent with the hypervirulence phenotype of KPB. These results demonstrate that even a low‐dose phage administration is sufficient to provide complete protection against an otherwise fatal infection.

Figure 5Therapeutic efficacy of phage vB_KpnP_B1 against KPB infection in mice. (A) Schematic of the experimental design for in vivo phage therapy. Mice received intraperitoneal injection of KPB (2.0 × 10^4^ CFU) at 0 h, followed by phage vB_KpnP_B1 (2.0 × 10^1^ PFU) at 2 h. Survival rates were monitored for 168 h (7 days). (B) Survival curves of mice treated with PBS, KPB alone, or KPB plus phage. All mice succumbed to infection within 24 h in the KPB group, whereas phage treatment significantly improved survival (*n* = 6 per group). (C) Representative H&E stained sections of heart, liver, spleen, lung, and kidney tissues from mice infected with strain KPB, phage‐treated group and PBS–treated control. Severe inflammatory infiltration and tissue damage were observed in the KPB–infected group, which were markedly alleviated in phage‐treated mice (scale bars, 100 μm).

**Figure:**
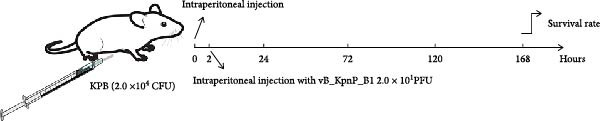
(A)

**Figure:**
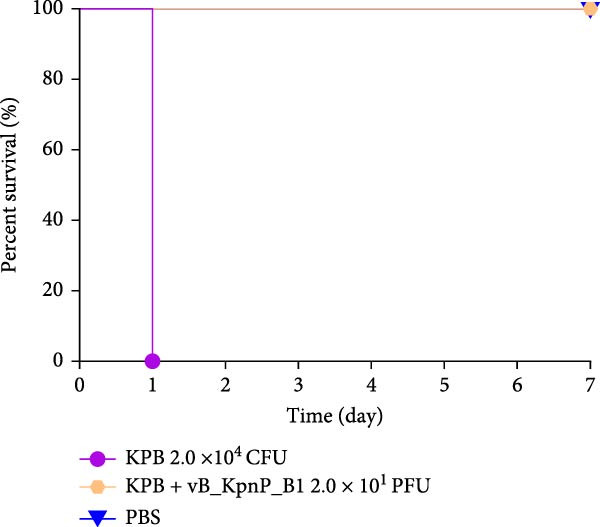
(B)

**Figure:**
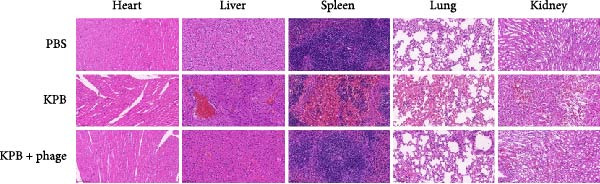
(C)

Histopathological examination of major organs further supported the therapeutic efficacy of phage treatment (Figure 5C). PBS–treated mice displayed severe pathological lesions, including extensive necrosis, hemorrhage, and dense inflammatory infiltrates in the heart, liver, spleen, lungs, and kidneys—indicative of uncontrolled systemic dissemination. In contrast, phage‐treated animals maintained near‐physiological tissue architecture, with minimal inflammatory changes and no observable necrosis in key target organs, such as the liver and lungs. These findings suggest that phage therapy not only prevents host mortality but also mitigates organ‐specific damage associated with a KPB infection.

Together, these data highlight the robust in vivo efficacy of phage vB_KpnP_B1 in controlling HvKp infection, offering both systemic protection and tissue‐level preservation. This supports the potential application of phage therapy as a precision antimicrobial strategy against multidrug‐resistant pathogens.

## 4. Discussion

Our findings unveiled the ST25 lineage as a notable hypervirulent *K. pneumoniae* clone capable of inflicting severe systemic disease across mammalian hosts, with significant zoonotic and nosocomial transmission potential. ST25 exhibits a hybrid pathogenic profile: It carries key hypervirulence determinants typical of classical community‐associated HvKp lineages but spans community/hospital settings and multiple hosts while harboring diverse plasmids and carbapenemases that link it to MDR high‐risk clones. The phylogenetic clustering of ST25 isolates from geographically distant regions, coupled with conserved virulence genotypes in clinical strains, suggests that this lineage has undergone recent clonal expansion through adaptive evolution rather than sporadic genetic exchange. Core‐genome phylogeny of 291 ST25/ST11/ST23 isolates confirms these three lineages form distinct, independently evolved clades; notably, the swine‐derived KPB isolate clusters tightly with human clinical ST25 strains, reinforcing cross‐host circulation potential and zoonotic transmission risk. Notably, the dominance of ST25 in East Asian clinical settings aligns with prior reports of hypervirulent *K. pneumoniae* epidemics in this region [5, 43], but our identification of ST25 in livestock and healthcare environments implies a broader ecological niche than previously recognized.

The pathogenicity of ST25 is mechanistically anchored in its dual dependence on CPS biosynthesis and high‐affinity iron acquisition systems. Both *wcaJ*/*wzc* and *rmpA* emerged as nonredundant virulence determinants, with their disruption eliminating lethality in mouse models. The distinct attenuation of capsule‐deficient mutants also manifested in significantly reduced adhesion to epithelial cells and heightened susceptibility to phagocytosis, highlighting the capsule’s multifaceted role in immune evasion and host colonization. This aligns with the established role of CPS in immune evasion [48], but our data uniquely demonstrate that a swine‐derived ST25 strain exhibited a capsular architecture indistinguishable from human clinical isolates, suggesting shared virulence strategies across hosts. Furthermore, the reduced mortality of iron acquisition mutants (Δ*iroB* and Δ*iucABCD-iutA*) highlights the critical role of iron scavenging in acute infection—a finding congruent with studies linking aerobactin to hypervirulence in human‐associated strains [49, 50]. However, the retention of partial lethality in these mutants suggests compensatory iron uptake mechanisms, possibly via redundant siderophores or host iron mobilization pathways.

The systemic multiorgan pathology observed in swine mirrors the disseminated infection patterns of HvKp in humans, validating this model for studying host‐pathogen interactions in a physiologically relevant context. Importantly, the rapid clinical progression and histopathological features (e.g., hemorrhagic lymphadenitis and interstitial nephritis) observed here correlate with hypervirulence markers, reinforcing the translational relevance of these models for therapeutic development.

Beyond pathogenicity mechanisms, our work introduced a promising therapeutic approach through phage vB_KpnP_B1, which resulted in 100% survival in preclinical models despite delayed administration. This efficacy parallels recent successes in phage therapy against carbapenem‐resistant *K. pneumoniae* [34, 51, 52]. However, this study is the first to demonstrate its applicability to hypervirulent zoonotic strains. The absence of residual tissue damage in phage‐treated animals underscores the potential of phage therapy to mitigate both microbial burden and immune‐mediated pathology—a dual advantage over conventional antibiotics. In addition to these findings, accumulating evidence supports the safety and practical utility of bacteriophages in livestock. Previous studies in pigs, cattle, and poultry have demonstrated that therapeutic phages are generally well tolerated, nontoxic, and highly specific, with minimal disruption to the resident microbiota [53, 54]. Moreover, controlled trials have shown that phage therapy can markedly reduce bacterial burdens in livestock, underscoring its potential as an alternative antimicrobial strategy in agricultural settings [55]. Together with our demonstration of complete protection in murine models, these data highlight the translational promise of vB_KpnP_B1 for future applications within a One Health framework.

## Ethics Statement

This study was conducted in accordance with the ARRIVE (Animal Research: Reporting of In Vivo Experiments) guidelines. The animal experiments and all cell‐based assays were conducted in accordance with the guidelines of Henan Agricultural University and were approved by the Animal Care Committee of Henan Agricultural University (HNND2021071401).

## Disclosure

All authors have read and agreed to the published version of the manuscript.

## Conflicts of Interest

The authors declare no conflicts of interest.

## Author Contributions

Conceptualization: **Xiang-Dang Du** and **Chunyan Xu**. Methodology and investigation: **Zheng Chen**, **Yang Liu**, **Congyang Du**, **Shengguo Gao**, **Lufeng Zhai**, **Weicheng Liu**, **Qiong Li**, **Xue Liu**, and **Yuzhe Zhao**. Validation: **Zheng Chen** and **Xiang-Dang Du**. Software: **Runhao Yu** and **Longyu Li**. Writing – original draft preparation: **Zheng Chen**, **Xiang-Dang Du**, and **Chunyan Xu**. Writing – review and editing: **Xiang-Dang Du**, **Chunyan Xu**, **Xue Liu**, and **Stefan Schwarz**. Writing – supervision and funding acquisition: **Hong Yao**, **Lei Luo**, **Chunyan Xu**, and **Xiang-Dang Du**. Zheng Chen and Yang Liu have contributed equally to this work.

## Funding

This study was supported by the National Key Research and Development Program of China (2021YFD1800900).

## Supporting Information

Additional supporting information can be found online in the Supporting Information section.

## Supporting information


**Supporting Information 1** Table S1: The sequence information used for the deletion mutants constructed in this study. Tables S2 and S3: Core genome phylogenetic analysis of bacterial strains used in this study.

**Supporting Information 2** Figure S1: Comparison of the genetic contexts harboring virulence genes between the two plasmids pKPB_Vir and pNTUH‐K2044.

**Supporting Information 3** Figure S2: Phylogenetic analysis of 291 *K.pneumoniae* strains representing ST11, ST25, and ST23 sequence types.

## Data Availability

The datasets generated and analyzed during this study are available from the corresponding author on reasonable request.
