# Supplementary figures and images for: Evolutionary Origins and Virulence Determinants of ST25 Hypervirulent Klebsiella pneumoniae in Swine: Genomic Insights and Functional Validation
Source: Transbound Emerg Dis. 2026 Jan 21;2026:4488875. doi: 10.1155/tbed/4488875 (PMC12820796; doi:10.1155/tbed/4488875)

## Slide 1
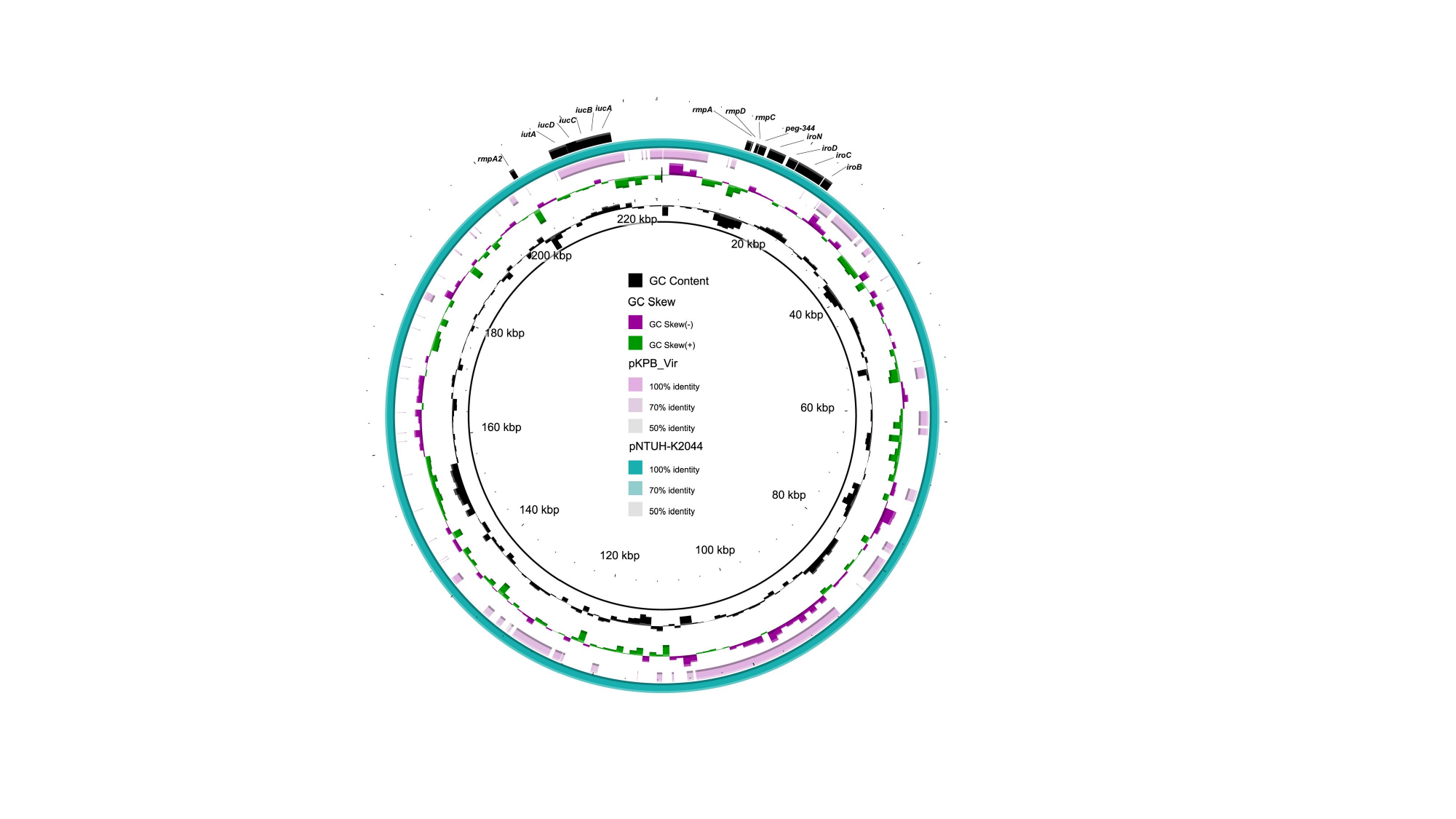

Supplement: Supplementary file 2 — Supporting Information 2 Figure S1: Comparison of the genetic contexts harboring virulence genes between the two plasmids pKPB_Vir and pNTUH‐K2044. [file TBED-2026-4488875-s003.pptx]

## Slide 1
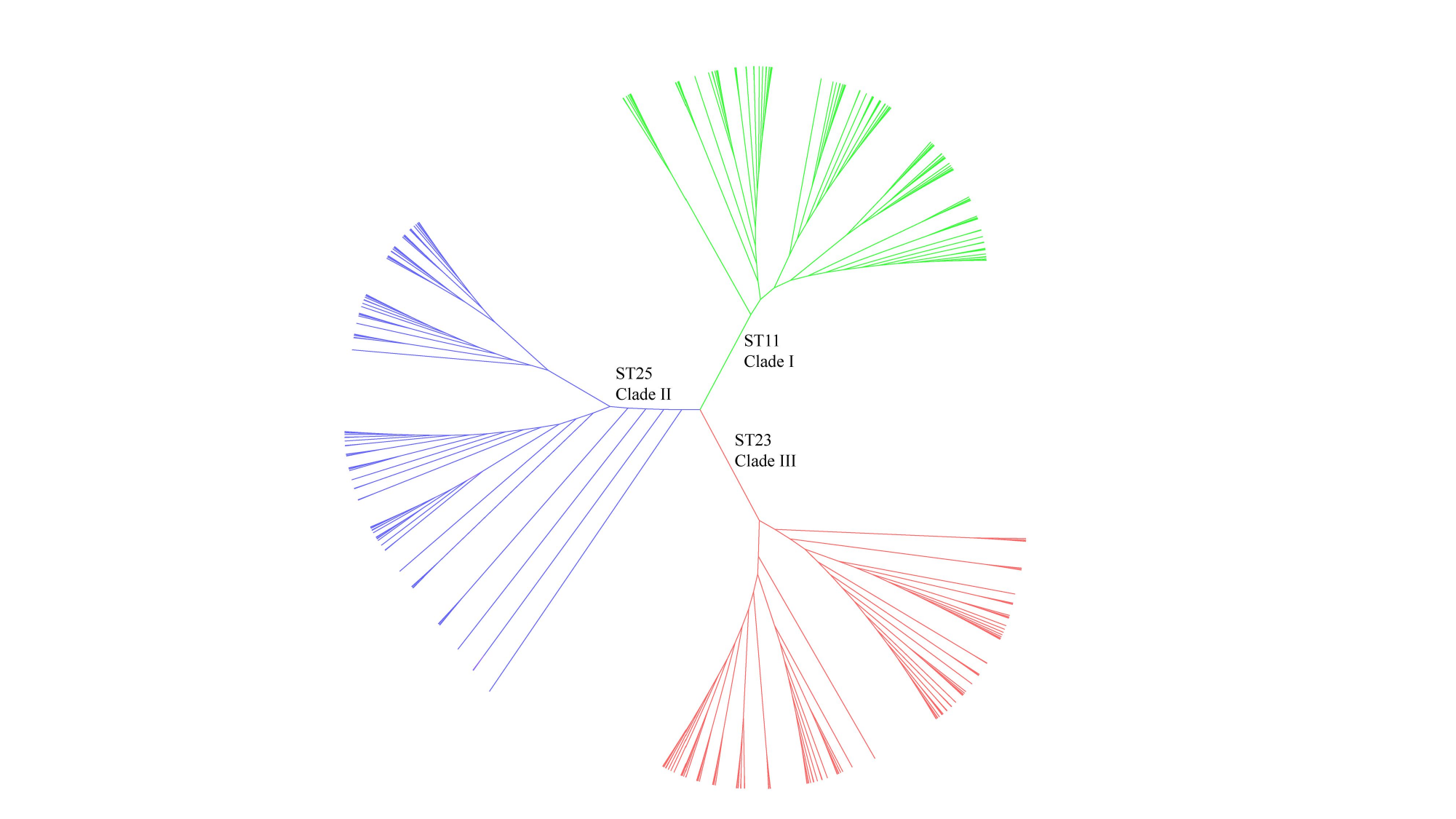

Supplement: Supplementary file 3 — Supporting Information 3 Figure S2: Phylogenetic analysis of 291 K.pneumoniae strains representing ST11, ST25, and ST23 sequence types. [file TBED-2026-4488875-s002.pptx]
